# Global Distribution of Invasive Serotype 35D Streptococcus pneumoniae Isolates following Introduction of 13-Valent Pneumococcal Conjugate Vaccine

**DOI:** 10.1128/JCM.00228-18

**Published:** 2018-06-25

**Authors:** Stephanie W. Lo, Rebecca A. Gladstone, Andries J. van Tonder, Paulina A. Hawkins, Brenda Kwambana-Adams, Jennifer E. Cornick, Shabir A. Madhi, Susan A. Nzenze, Mignon du Plessis, Rama Kandasamy, Philip E. Carter, Özgen Köseoglu Eser, Pak Leung Ho, Naima Elmdaghri, Sadia Shakoor, Stuart C. Clarke, Martin Antonio, Dean B. Everett, Anne von Gottberg, Keith P. Klugman, Lesley McGee, Robert F. Breiman, Stephen D. Bentley

**Affiliations:** a Infection Genomics, The Wellcome Trust Sanger Institute, Wellcome Trust Genome Campus, Hinxton, Cambridge, United Kingdom; b Hubert Department of Global Health, Rollins School of Public Health, Emory University, Atlanta, Georgia, USA; c Respiratory Diseases Branch, Centers for Disease Control and Prevention, Atlanta, Georgia, USA; d Vaccines and Immunity Theme, Medical Research Council Unit The Gambia, Banjul, Fajara, The Gambia; e Malawi Liverpool Wellcome Trust Clinical Research Programme, Blantyre, Malawi; f Institute of Infection & Global Health, University of Liverpool, Liverpool, United Kingdom; g Medical Research Council: Respiratory and Meningeal Pathogens Research Unit, University of the Witwatersrand, Johannesburg, South Africa; h Department of Science and Technology/National Research Foundation: Vaccine Preventable Diseases, University of the Witwatersrand, Johannesburg, South Africa; i Centre for Respiratory Disease and Meningitis, National Institute for Communicable Diseases of the National Health Laboratory Service, Johannesburg, South Africa; j School of Pathology, University of the Witwatersrand, Johannesburg, South Africa; k Oxford Vaccine Group, Department of Paediatrics, University of Oxford, and the NIHR Oxford Biomedical Research Centre, Oxford, United Kingdom; l Institute of Environmental Science and Research Limited, Kenepuru Science Centre, Porirua, New Zealand; m Hacettepe University Faculty of Medicine, Department of Medical Microbiology, Ankara, Turkey; n Department of Microbiology and Carol Yu Centre for Infection, The University of Hong Kong, Queen Mary Hospital, Hong Kong, China; o Department of Microbiology, Faculty of Medicine and Pharmacy, Hassan II University of Casablanca, Casablanca, Morocco; p Bacteriology-Virology and Hospital Hygiene Laboratory, University Hospital Centre Ibn Rochd, Casablanca, Morocco; q Department of Pathology and Laboratory Medicine and Department of Paediatrics and Child Health, The Aga Khan University, Karachi, Pakistan; r Faculty of Medicine and Institute of Life Sciences, University of Southampton, Southampton, United Kingdom; s Microbiology and Infection Unit, Warwick Medical School, Warwick, United Kingdom; t London School of Hygiene and Tropical Medicine, London, United Kingdom; u University of Edinburgh, The Queens Medical Research Institute, Edinburgh, United Kingdom; v Emory Global Health Institute, Emory University, Atlanta, Georgia, USA; University of Iowa College of Medicine

**Keywords:** 35D, PCV, novel serotype, whole-genome sequencing

## Abstract

A newly recognized pneumococcal serotype, 35D, which differs from the 35B polysaccharide in structure and serology by not binding to factor serum 35a, was recently reported. The genetic basis for this distinctive serology is due to the presence of an inactivating mutation in *wciG*, which encodes an O-acetyltransferase responsible for O-acetylation of a galactofuranose. Here, we assessed the genomic data of a worldwide pneumococcal collection to identify serotype 35D isolates and understand their geographical distribution, genetic background, and invasiveness potential. Of 21,980 pneumococcal isolates, 444 were originally typed as serotype 35B by PneumoCaT. Analysis of the *wciG* gene revealed 23 isolates from carriage (*n* = 4) and disease (*n* = 19) with partial or complete loss-of-function mutations, including mutations resulting in premature stop codons (*n* = 22) and an in-frame mutation (*n* = 1). These were selected for further analysis. The putative 35D isolates were geographically widespread, and 65.2% (15/23) of them was recovered after the introduction of pneumococcal conjugate vaccine 13 (PCV13). Compared with serotype 35B isolates, putative serotype 35D isolates have higher invasive disease potentials based on odds ratios (OR) (11.58; 95% confidence interval[CI], 1.42 to 94.19 versus 0.61; 95% CI, 0.40 to 0.92) and a higher prevalence of macrolide resistance mediated by *mefA* (26.1% versus 7.6%; *P* = 0.009). Using the Quellung reaction, 50% (10/20) of viable isolates were identified as serotype 35D, 25% (5/20) as serotype 35B, and 25% (5/20) as a mixture of 35B/35D. The discrepancy between phenotype and genotype requires further investigation. These findings illustrated a global distribution of an invasive serotype, 35D, among young children post-PCV13 introduction and underlined the invasive potential conferred by the loss of O-acetylation in the pneumococcal capsule.

## INTRODUCTION

Streptococcus pneumoniae (pneumococcus) is an important human pathogen that causes pneumonia, bacteremia, and meningitis. In 2015, >330,000 deaths globally in children of <5 years old were estimated to have been caused by pneumococci (1). The polysaccharide capsule of pneumococcus, which has almost 100 serological variants (serotypes), is a major virulence factor (2, 3). Pneumococcal conjugate vaccines (PCVs) targeting up to 13 serotypes have gradually been introduced into 139 countries since the early 2000s (http://view-hub.org/viz/). Simultaneously, a proportional increase in nonvaccine serotypes, such as serotype 35B, has been reported in various countries (4).

Recently, a serotype 35B variant, 35D, was identified in four pneumococcal isolates in Australia (5) and two in the United States (2, 6), all of which had an inactivating mutation in *wciG*, which encodes an O-acetyltransferase responsible for O-acetylation of a galactofuranose. Nuclear magnetic resonance (NMR) analysis on a single isolate representing this novel pneumococcal serotype verified that the serotype 35D capsule lacked O-acetylation but that it was otherwise identical to serotype 35B (2). Serologically, serotype 35D is distinct from serotype 35B by consistently not binding to factor serum 35a, but it displays variable reactivity to group 35 antiserum (2, 5, 6). WciG functionality has been shown to be the determinant of factor serum 35a recognition (2, 7).

Presence and absence of O-acetylation is one of the mechanisms for generating diversity in capsular structure, as shown by other serotype pairs such as 9V/9A (O-acetylation mediated by WciE) (8), 11A/11E (WcjE) (8), 15B/15C (WciZ) (8), 33A/33F (WcjE) (9), and 35C/42 (WciG) (7). It is noteworthy that the O-acetyl group in the capsular repeat unit is important for innate immune recognition (10) and is the target of vaccine-elicited antibodies (11). Loss of O-acetylation in serotype 11E is predicted to assist pneumococci in evading host immune and vaccine response and has been suggested to occur during invasive disease after initial colonization with the serotype 11A strain expressing an O-acetylated form of capsule (12). The role of loss of O-acetylation in pneumococcal survival during invasion among the other serotype pairs has remained unknown due to the rarity of serotypes 9A, 33A, and 42 for comparisons, and by the difficulty in differentiation between serotype 15B and 15C.

Although the serological profile and biochemical structure of serotype 35D have been described, there has not been an opportunity to comprehensively study this serotype across geographies and clinical considerations. Here, we assessed the genomic data on serotype 35D isolates from a worldwide pneumococcal collection to understand this serotype's geographical distribution, genetic background and potential invasiveness.

## MATERIALS AND METHODS

We retrospectively determined serotypes of 21,980 assembled pneumococcal genomes from the Global Pneumococcal Sequencing (GPS) project (*n* = 16,575; May 2017; http://www.pneumogen.net/gps/) and a compiled data set (*n* = 5,405) by van Tonder et al. (13). DNA extraction was performed on a pure overnight culture derived from a single colony. Sequencing was performed on the Illumina HiSeq platform to produce paired-end reads of either 75 (in 2010 and 2011), 100 (in 2013 and 2014), or 125 (in 2015 and 2016) bp in length. *In silico* serotype was determined using the whole-genome sequence (WGS)-based serotyping method PneumoCaT (14). As the current version of PneumoCaT does not distinguish serotype 35D from serotype 35B, all samples that were initially typed as serotype 35B were included in this study. To differentiate these two serotypes, nucleotide sequences of *wciG* were extracted from the assembled genome sequences and aligned to a reference sequence of 35B *wciG* (GenBank accession number KX021817) described by Geno et al. (2) using CLUSTALW (15). Nonsense and frameshift mutations that led to premature stop codons and in-frame insertions/deletions in *wciG* were predicted to result in complete loss of function and reduction of function of the WciG protein, respectively. Isolates with these mutations were *in silico* typed as serotype 35D, and their phenotypic serotype were determined by the Quellung reaction, tested on an overnight culture derived from a single colony (16). Phylogenetic analysis was performed on all serotype 35B and 35D isolates by constructing a maximum likelihood tree using RAxML v.8.2.X (17) based on single-nucleotide polymorphism sites extracted from a core gene alignment with Roary v.3.6.1 (18). An empirical odds ratio for invasive disease due to serotype 35B and 35D was calculated based on a pneumococcal collection of 3,333 randomly selected carriage (*n* = 1,260) and disease (*n* = 2,073) isolates from children aged <2 years old, collected during the pre-PCV (*n* = 1,691), post-PCV7 (*n* = 678), and post-PCV13 (*n* = 964) eras using a previously described method (19). For each country, the random selection was carried out from a collection of disease isolates collected via laboratory-based surveillance and carriage isolates collected via cohort studies using the following criteria: 50% of the isolates represented the pre-PCV period (≤1 year before) and 50% the post-PCV period (≥2 years after primary and ≥1 after subsequent PCVs). The randomly selected collection in this study included 67 different serotypes plus nontypeable pneumococci. These isolates were collected in South Africa (carriage *n* = 721, disease *n* = 1,047), Malawi (carriage *n* = 336, disease *n* = 60), and the Gambia (carriage *n* = 1,016, disease *n* = 153). Isolates from other locations in the GPS data set were either not randomly selected or consisted of only disease or only carriage isolates and thus could not be used to calculate odds ratios. Susceptibility to chloramphenicol, co-trimoxazole, erythromycin, penicillin, and tetracycline were predicted by the identification of resistant determinants in the assembled genomes using previously described pipelines (20–22). The epidemiological and phylogenetic data can be interactively visualized and analyzed online by using the Microreact tool (https://microreact.org/project/GPS_serotype_35B_35D).

## RESULTS AND DISCUSSION

Of 21,980 assembled pneumococcal genomes from the Global Pneumococcal Sequencing (GPS) project (*n* = 16,575; May 2017) and a compiled data set (*n* = 5,405) by van Tonder et al. (13), 444 isolates from disease (*n* = 173), carriage (*n* = 270), and an unknown source (*n* = 1) were originally typed as serotype 35B by PneumoCaT (5). The *wciG* alignment revealed that 78.6% (349/444) of isolates were identical to the serotype 35B reference, 8.3% (37/444) had silent mutations, 7.9% (35/444) had missense mutations, 3.4% (15/444) had frameshift mutations, 1.6% (7/444) had nonsense mutations, and 0.2% (1/444) had an in-frame insertion. All frameshift mutations led to a premature stop codon that disrupted the coding region of *wciG*. Given that the latter three types of mutations lead to reduced function or a complete loss of function of WciG, the 23 isolates were designated serotype 35D (Table 1). The Quellung reaction of 20 viable isolates showed that 50% (10/20) were serologically typed as serotype 35D, 25% (5/20) as serotype 35B, and 25% (5/20) as a mixture of serotype 35B and 35D (Table 2). In all discrepant cases, we examined the *cps* locus sequences in an attempt to identify any gene loss and mixed *wciG* alleles. The *cps* locus region shared the same capsular genes with the serotype 35D reference (GenBank accession number KY084476), and the mutations in *wciG* were supported by at least 42× depth of reads (median, 80×; range, 42× to 143×) with 100% consistency. The discrepancy between phenotype and genotype could be due to (i) our inability to capture the serotype diversity in a clinical sample, since the bacterial cultures subjected to DNA extraction and Quellung testing were derived from a single colony that could be different between experiments, and (ii) the possible interconvertibility between serotype 35B and 35D during bacterial culture *in vitro*. In all five isolates that were both positive and negative to antisera fs35a under one microscope (Table 2), the mutations in *wciG* were either a 1-bp insertion or deletion that occurred after a 6- to 7-bp homopolymer, highlighting the possibility of interconversion between serotype 35B and 35D during DNA replication. Metagenomic analysis of clinical samples to snapshot the serotype diversity and investigation into the interconvertibility of serotype 35B and 35D will potentially explain the discrepancy between the phenotypes and genotypes observed in this study. Considering the limitation of this study and our recent understanding of the genetic basis that differentiates serotype 35B and 35D (2, 6, 7), the nonsilent mutations detected in *wciG* in this study strongly indicated the presence of serotype 35D pneumococci in the sample. Thus, the 23 *in silico* serotype 35D isolates were selected for further analysis.

**TABLE 1:** Genetic diversity of inactivating mutations in *wciG* of 29 serotype 35D S. pneumoniae isolates from the Global Pneumococcal Sequencing (GPS) project (*n* = 23) and previous studies (*n* = 6)

Type of mutation (*n*) or *wciG* nucleotide mutation	*n*	Clonal complex and or sequence type (*n*)	Isolation:			Reference or source
			Geographical location(s) (*n*)	Yr(s) (*n*)	Site(s)^*e*^ (*n*)	
Frameshift mutation (18)^*a*^						
86_87insG	6	CC156 (2), CC558 (2), CC198 (1), CC9813 (1)	Malawi (2), New Zealand (1), Senegal (1), South Africa (1), United States (1)	2006 (1), 2011 (1), 2012 (2), 2015 (2)	CSF (3), blood (2), joint pus (1)	GPS
914_929del_16bp	2	CC558	South Africa, United States	2012 (1), 2013 (1)	CSF (1), blood (1)	GPS
162_163insT	2	CC558	United States	2004 (1), 2007 (1)	Nasopharynx (2)	GPS^*d*^
92_93insC	1	CC198	The Gambia	2013	Blood	GPS
705_706insT	1	CC156	Malawi	2015	CSF	GPS
86delG	1	CC156	Cameroon	2012	CSF	GPS
312delA	1	CC198	The Gambia	2009	Nasopharynx	GPS
382_385_del_4bp	1	CC9813	South Africa	2012	CSF	GPS
306_307insA	1	CC198	Australia	2016	Unknown	5
36delA	1	CC558	Australia	2015	Unknown	5
663_696del_34bp	1	CC452	Australia	2016	Unknown	5
In-frame deletion/insertion (3)						
792_968del_177bp^*b*^	1	CC156	USA	2015	Blood (2)	6
755_808del_54bp^*b*^	1	CC558	Australia	2016	Unknown	5
523_524ins_15bp	1	CC558	USA	2009	Blood	GPS
Nonsense mutation (7)						
C220T	2	CC156, ST373	Nepal, South Africa	2013 (1), 2014 (1)	CSF (1), nasopharynx (1)	GPS
T732G	2	CC198	The Gambia	2014 (2)	CSF (1), blood (1)	GPS
C104A	1	CC558	USA	2012	Blood	GPS
C323A	1	CC558	USA	2012	Blood	GPS
T434G	1	CC198	The Gambia	2009	Lung aspirate	GPS
Missense mutations (1)						
G533A, G679A^*c*^	1	Unknown	USA	Unknown	Unknown	(2)

a All frameshift mutations resulted in a premature stop codon.

b The in-frame deletion rendered WciG, an acetyltransferase, nonfunctional. It was evidenced by the serological profiles reported by Chochua et al. (6) and Staples et al. (5).

c The resulting amino acid changes were R178K and A227T. The substitution led to a nonfunctional WciG, confirmed by serological test and NMR spectroscopic analysis.

d These two isolates were reported in a previous study by Croucher et al. (23) and *in silico* serotype was updated as serotype 35D in this study.

e CSF, cerebrospinal fluid.

**TABLE 2:** Serological profiles of 29 serotype 35D S. pneumoniae isolates from the Global Pneumococcal Sequencing (GPS) project (*n* = 23) and previous studies (*n* = 6) tested by Quellung reaction

Strain name	Country	CC	Yr	*wciG* mutation(s)^*f*^	Pool G	Type		Group 35	Antiserum					Phenotypic serotype	Reference or source
						29	42		fs35a	fs35b	fs35c	fs29b	fs42a		
3431-06	USA	N/A	N/A	G533A, G679A	+	ND	ND	−	−	−	+	+	−	35D	2
16S471	Australia	CC198	2016	306_307insA	+	+	+	+	−	−	+	+	−	35D	5
SAMDU-00005305	Australia	CC558	2015	36delA	+	+	+	+	−	−	+	+	−	35D	5
16S49	Australia	CC452	2016	663_696del_34bp	+	+	+	+	−	−	+	+	−	35D	5
16S35	Australia	CC558	2016	755_808del_54bp	+	+	+	+	−	−	+	+	−	35D	5
20152877	USA	CC156	2015	792_968del_177bp	+	ND	ND	+	−	−	+	+	−	35D	6
CH2075	USA	CC558	2007	162_163insT	+	+	−	+	+	−	+	+	−	35B	GPS^*e*^
3025	USA	CC558	2004	162_163insT	+	+	−	+	+	−	+	+	−	35B	GPS^*e*^
GPS_US_2010209945_R1	USA	CC558	2009	523_524ins_15bp	+	+	−	+	+	−	+	+	−	35B	GPS
GPS_GM_1130	The Gambia	CC198	2014	T731G (L244*)	+	+	−	+	+	−	+	+	−	35B	GPS
GPS_GM_1148	The Gambia	CC198	2014	T731G (L244*)	+	+	−	+	+	−	+	+	−	35B	GPS
GPS_ZA_2370	South Africa	CC9813	2012	382_385delATAT	+	+	+	+	−	−	+	+	−	35D	GPS
GPS_ZA_2636	South Africa	CC558	2013	914_929del_16bp	+	+	+	+^*b*^	−	−	+	+	−	35D	GPS
2012215593	USA	CC558	2012	914_929del_16bp	+	+	−	−	−	−	+	+	−	35D	GPS
2012215608	USA	CC558	2012	C104A (S35*)	+	+	−	−	−	−	+	+	−	35D	GPS
GPS_ZA_2559	South Africa	CC156	2013	C220T (Q74*)	+	+	+	+	−	−	+	+	−	35D	GPS
GPS_NP_7242	Nepal	Singleton^*d*^	2014	C220T (Q74*)	+	+	ND	+	−	−	+	+	−	35D	GPS
2012220613	USA	CC558	2012	C323A (S108*)	+	+	−	−	−	−	+	+	−	35D	GPS
2013208723	USA	CC558	2012	86_87insG	+	+	−	−	−	−	+	+	−	35D	GPS
GPS_MW_D38253_R1	Malawi	CC156	2006	86_87insG	+	+	−	−	−	−	+	+	−	35D	GPS
GPS_MW_BKR609	Malawi	CC156	2015	86_87insG	+	+	−	−	−	−	+	+	−	35D	GPS
PI0167	Senegal	CC198	2011	86_87insG	+	+	−	+	+^*b*^	−	+	+	−	35B/D	GPS
GPS_NZ_15SP0720	New Zealand	CC558	2013	86_87insG	+	+	ND	+	+^*c*^	−	+	+	−	35B/D	GPS
GPS_ZA_2487	South Africa	CC9813	2012	86_87insG	+	+	+	+	+^*b*^	−	+	+	−	35B/D	GPS
GPS_MW_BKR5WC	Malawi	CC156	2015	705_706insT	+	+	−	+^*b*^	+^*b*^	−	+	+	−	35B/D	GPS
PI0258	Cameroon	CC156	2012	86delG	+	+	−	+	+^*b*^	−	+	+	−	35B/D	GPS
GPS_GM_0282	The Gambia	CC198	2013	92_93insC	ND^*a*^	ND	ND	ND	ND	ND	ND	ND	ND	ND	GPS
GPS_GM_0600	The Gambia	CC198	2009	312delA	ND	ND	ND	ND	ND	ND	ND	ND	ND	ND	GPS
GPS_GM_0320	The Gambia	CC198	2009	T434G (L145*)	ND	ND	ND	ND	ND	ND	ND	ND	ND	ND	GPS

a ND, data not available.

b Under the microscope, cells that were derived from a single-colony overnight culture showed both positive and negative to the antisera tested.

c This isolate was tested in two different laboratories and exhibited as both positive to antiserum fs35a in one laboratory and negative in another.

d Isolate GPS_NP_7242 belong to ST373, a singleton that does not belong to any clonal complex.

e These two isolates were reported in a previous study by Croucher et al. (23) and *in silico* serotype was updated as serotype 35D in this study.

f *, stop codon.

**FIG 1:**
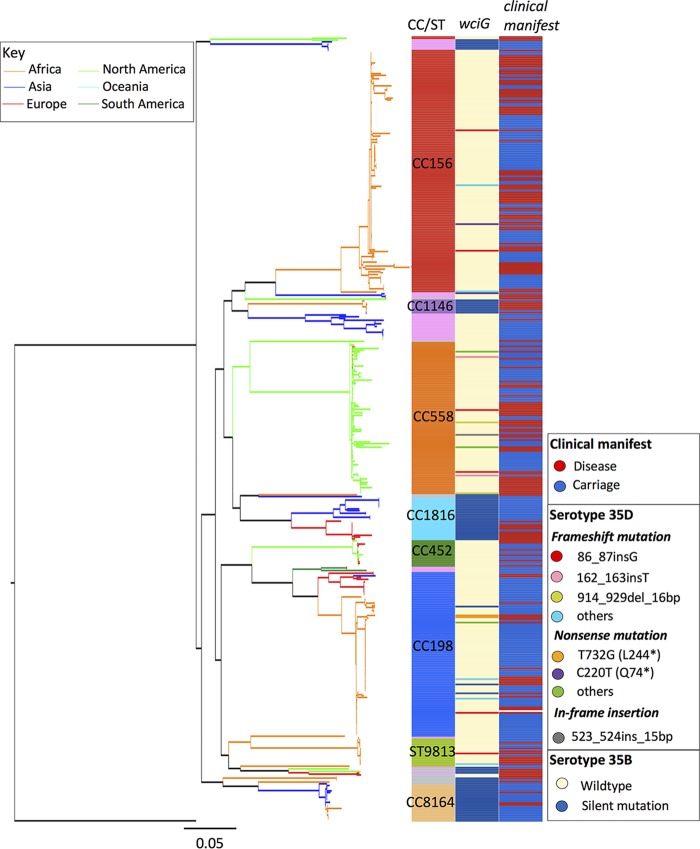
Maximum likelihood phylogenetic tree was constructed using 56,848 single-nucleotide polymorphisms (SNPs) extracted from a 1.02-Mb codon alignment of 1,141 core genes from 444 serotype 35B and 35D S. pneumoniae isolates. The tree is colored according to the geographic location of each sample's isolation. This analysis used an unrelated nontypeable isolate as the outgroup on which to root the tree. Clonal complex (CC) and mutations in *wciG* are shown to the right of the tree. Singleton sequence types and minor CCs with <5 isolates in this study are indicated in pink and gray, respectively.

The mutation patterns of *wciG* among the *in silico* serotype 35D isolates were diverse. The *wciG* mutation patterns in the 23 serotype 35D isolates were different from those of the 6 serotype 35D isolates reported previously (2, 5, 6). In total, there were 20 mutation patterns observed in 29 serotype 35D isolates from 10 countries across four continents (Table 1). The most common naturally deficient WciG was due to 86_87insG, which occurred within a 6-bp homopolymeric stretch of guanine. It was first observed in an isolate from Malawi in 2006, prior to the introduction of PCV7, and was also found in isolates from Senegal in 2011, South Africa and the United States in 2012, and New Zealand in 2015. Isolates with this mutation were sporadically distributed on the phylogenetic tree (Fig. 1), suggesting that the mutations had arisen independently on multiple occasions. The convergence of mutations may imply that this site is a mutational hot spot.

The majority of serotype 35D isolates belonged to clonal complex 558 (CC558) (*n* = 9), CC198 (*n* = 6), and CC156 (*n* = 5), which were primarily associated with serotype 35B (6, 24, 25). The CC558 and CC156 lineages accounted for most of the increase in serotype 35B isolates after the introduction of PCV13 in the United States (6), while CC198 is the major serotype 35B lineage in the Gambia (unpublished data). Based on a high-resolution single-nucleotide polymorphic tree (Fig. 1), serotype 35D pneumococci emerged among closely related serotype 35B isolates within different clusters. Together with the unrelated mutations observed in *wciG*, this strongly indicated that serotype 35B is the progenitor of serotype 35D.

Compared with serotype 35B isolates, serotype 35D isolates were more likely to be recovered from sterile anatomical sites, including cerebrospinal fluid (CSF; *n* = 9), blood (*n* = 8), lung aspirate (*n* = 1), and joint aspirate (*n* = 1), than among carriage isolates (*n* = 4) (82.6% [19/23] versus 36.7% [154/420]; *P* < 0.001 by Fisher's exact test). Based on a larger pneumococcal collection (*n* = 3,333) randomly selected from the GPS project database, the empirical odds ratio (OR) for invasive disease due to serotype 35D is 11.58 (95% confidence interval, 1.42 to 94.19), whereas the OR for serotype 35B is 0.61 (95% CI, 0.40 to 0.92). The increased invasive capacity in serotype 35D strains could be a result of evasion of the immune response targeting the capsule O-acetyl group. The observation in serotype 35B/35D coincides with a previous study on serotype 11A/11E, in which serotype 11E strains with a loss or reduced amount of acetylation in the capsule were found to be significantly associated with invasive pneumococcal disease (12, 26). The emergence of serotype 35D is likely explained by Calix et al.'s hypothesis (12) that pneumococcal capsule structure undergoes microevolution during progression from carriage to infection in response to divergent selection pressure in early mucosal colonization compared to later in a sterile site. This model of microevolution needs to be further investigated by characterizing the serotype dynamic over the development of invasive disease *in vivo*.

Compared with the pre-PCV era, the prevalence of serotype 35D has not increased more than serotype 35B after the introduction of PCV13. (OR, 12.36; 95% CI, 1.5 to 100.6 versus OR, 3.54; 95% CI, 2.4 to 5.4; Table 3) in the randomly selected pneumococcal collection. A large proportion of 35D isolates (65.2%, 15/23) were collected after the rollout of PCV13. The post-PCV introduction isolates were all invasive isolates and were recovered in six countries (Cameroon, Malawi, New Zealand, South Africa, the Gambia, and the United States), highlighting that this invasive serotype is present in the residual pneumococcal population worldwide and could potentially be an example of serotype replacement.

**TABLE 3:** The prevalence of serotype 35B and 35D S. pneumoniae from South Africa (*n* = 1,768), the Gambia (*n* = 1,169) and Malawi (*n* = 396) in each vaccine period

Vaccine period^*a*^	No. of isolates (%) for serotype:		Odds ratio (95% confidence interval) for serotype:	
	serotype 35B	serotype 35D	35B	35D
Pre-PCV (*n* = 1691)	36 (2.12)	1 (0.06)	Baseline	Baseline
Post-PCV7 (*n* = 678)	12 (1.77)	0	0.83 (0.4–1.6)	
Post-PCV13 (*n* = 964)	69 (7.16)	7 (0.73)	3.54 (2.4–5.4)^*b*^	12.36 (1.5–100.6)^*b*^

a Based on the year of PCV introduction, we grouped each year of collection into three categories, as follows: pre-PCV period (years when no conjugated vaccine was used and the year of PCV7 introduction); post-PCV7 (the second year of PCV7 introduction until the year when a higher-valency PCV was introduced); and post-PCV13 (the second year of PCV13 introduction until the end of the study year). PCV7 was introduced in South Africa and the Gambia in 2009; PCV13 was introduced in South Africa, the Gambia, and Malawi in 2011.

b *P* value < 0.05.

Among the 23 serotype 35D isolates, 87.0% (20/23) had at least one resistance determinant conferring resistance to commonly used antibiotics, including penicillin (65.2%, 15/23), erythromycin (30.4%, 7/23), co-trimoxazole (21.7%, 5/23), and tetracycline (4.3%, 1/23). Similar to the previous studies on serotype 35B (6, 24), the penicillin-resistant isolates in this study were predominantly CC558 (60.0%, 9/15), followed by CC156 (35.7%, 5/15) and a singleton of sequence type 73 (ST373) (6.7%, 1/15). Macrolide resistance mediated by *mefA* was significantly higher in serotype 35D isolates than in serotype 35B isolates (Table 4). Five of six serotype 35D isolates harboring *mefA* were from the United States, where macrolides are recommended for use as an empirical therapy for pneumonia in children (27–29); they all belonged to CC558, a major contributor to penicillin resistance in the United States after introduction of PCV13 (24). Unlike the highly invasive but usually antibiotic-susceptible serotype 1, pneumococci expressing serotype 35B (lower-invasive capsule) are more likely to be commensal in the nasopharynx, which could allow them to acquire antibiotic resistance determinants via horizontal gene transfer from other nasopharyngeal bacteria; a subsequent switch to serotype 35D (high-invasive capsule) would then transform the antibiotic-resistant strain into a more virulent form.

**TABLE 4:** Antimicrobial resistant determinants in serotype 35B and 35D S. pneumoniae isolates from the Global Pneumococcal Sequencing (GPS) project

Antibiotic resistance determinant(s)	No. of isolates (%) for serotype:		*P* value
	35B (*n* = 421)	35D (*n* = 23)	
*ermB*	3 (0.7)	1 (4.3)	0.192
*mefA*	32 (7.6)	6 (26.1)	0.009
*tetM*	36 (8.6)	1 (4.3)	0.710
*folA* I100L and *folP* insertion	140 (33.3)	5 (21.7)	0.361

The limitation of this study is that the carriage and disease isolates included for calculating the invasiveness index were sampled in different cities in each country; all isolates included were collected between 2007 and 2015 from children aged <2 years old. Ideally, the carriage and disease isolates should be geography-, time-, and age-matched. In this instance, we calculated ORs for invasiveness separately for each country. The ORs for invasive disease due to serotype 35B and 35D in the Gambia were 0.37 (95% CI, 0.09 to 1.56) and 20.3 (95% CI, 2.10 to 196.42), respectively. The ORs could not be calculated for invasive disease, as all serotype 35D isolates in South Africa and Malawi were from disease. The ORs for disease due to 35B in South Africa and Malawi were 0.68 (95% CI, 0.40 to 1.16) and 0.72 (95% CI, 0.11 to 2.15), respectively. The ORs by country were consistent with the ORs calculated from the combined data sets of all three countries. Another limitation was that the effects of an in-frame insertion of 15 bp and the missense mutations in *wciG* on the protein function have not been evaluated. Removing these samples from all comparisons of serotype 35B and 35D did not alter the conclusions drawn from the statistical analyses.

This study highlighted the global distribution of an invasive serotype, 35D, among young children in the post-PCV13 era and underlined the invasive potential conferred by the loss of O-acetylation in the pneumococcal capsule.
